# Timing and Amount of Physical Therapy Treatment are Associated with Length of Stay in the Cardiothoracic ICU

**DOI:** 10.1038/s41598-017-17624-3

**Published:** 2017-12-14

**Authors:** Audrey M. Johnson, Angela N. Henning, Peter E. Morris, Alejandro G. Villasante Tezanos, Esther E. Dupont-Versteegden

**Affiliations:** 1 Department of Rehabilitation Sciences, College of Health Sciences, University of Kentucky, Lexington, Kentucky United States of America; 2 Rehabilitation Department, UK HealthCare, Lexington, Kentucky United States of America; 3 Department of Medicine, Division of Pulmonary, Critical Care and Sleep Medicine, Lexington, Kentucky United States of America; 4 Department of Statistics, College of Arts and Sciences, University of Kentucky, Lexington, Kentucky United States of America; 5 Center for Muscle Biology, College of Health Sciences, University of Kentucky, Lexington, Kentucky United States of America

## Abstract

Significant variability exists in physical therapy early mobilization practice. The frequency of physical therapy or early mobilization of patients in the cardiothoracic intensive care unit and its effect on length of stay has not been investigated. The goal of our research was to examine variables that influence physical therapy evaluation and treatment in the intensive care unit using a retrospective chart review. Patients (n = 2568) were categorized and compared based on the most common diagnoses or surgical procedures. Multivariate semi-logarithmic regression analyses were used to determine correlations. Differences among patient subgroups for all independent variables other than age and for length of stay were found. The regression model determined that time to first physical therapy evaluation, Charlson Comorbidity Index score, mean days of physical therapy treatment and mechanical ventilation were associated with increased hospital length of stay. Time to first physical therapy evaluation in the intensive care unit and the hospital, and mean days of physical therapy treatment associated with hospital length of stay. Further prospective study is required to determine whether shortening time to physical therapy evaluation and treatment in a cardiothoracic intensive care unit could influence length of stay.

## Introduction

Patients who require intensive critical care during hospitalization have high morbidity and mortality rates, experience decline in functional status and incur high costs of care overall^1^. Impaired exercise capacity, persistent weakness, enduring neuropsychological impairments and high costs of healthcare utilization in these patients lead to a negative post ICU trajectory attributed to muscle wasting and weakness^2–4^. The suboptimal quality of life survivors report is likely related to the significant physical, psychological, and financial complications that endure over time^5,6^. Early active mobility during ICU care is now recommended by International guidelines and is often performed by interprofessional teams consisting of physical therapists and other ICU care team members^7^. Early active mobilization includes active or resistive range of motion exercises, position changes in bed, transfers out of bed, progressive mobility protocols and ambulation; and increasingly studies show promising results^1,3,8–12^. More importantly, this clinical practice has been found to be safe^2,13,14^ and highlights the improvement early active mobilization produces in the re-acquisition of mobility skills^2,13^ although prospective studies have not shown reductions in length of stay^12,14–16^.

Despite growing support for the benefits of early active mobilization of patients in the ICU, significant variability in this practice exists^17^. Timing and amount of physical therapy during critical illness, staffing levels sufficient to meet patients’ needs, guidelines or protocols for recommended care, leadership, staff education, and variability in type of staff used during early mobility programs have been attributed to these differences^13,18–20^. Little is known about the practice of early active mobilization with physical therapists or physical therapist assistants in patients with cardiac or neurologic conditions despite higher ICU utilization by these patients^21^. Complications related to clinical care are a risk for cardiothoracic ICU patients, who are increasingly diagnosed with frailty^22–26^, which may predict post-surgical complications including, mortality, prolonged ventilation, and poor medical and functional outcomes^27,28^. These complications and comorbidities have the potential to influence time to physical therapy evaluation and consistency of physical therapy treatment to achieve early active mobilization of patients, but it is currently unknown what factors influence timing and amount of treatment. Understanding current mobilization practices with physical therapists and/or other members of the care team is critical to the design and implementation of strategies to improve the early active mobilization to the diverse population of critically ill patients in the ICU^17,29^.

Physical therapy evaluation and treatment in the acute care setting is highly focused on functional mobility status with interventions related to patient functional ability (bed mobility, transfers, use of assistive devices, gait or ambulation, etc.) representing the greatest percentage of treatment^30^. Both early mobilization and rehabilitation begin upon achievement of hemodynamic stability and international consensus on guidelines for safe mobilization of ventilated patients was achieved using criteria established for a variety of medical considerations^31–34^. These considerations can be a barrier to initiation of physical therapy evaluation and consistent performance of physical therapy treatment sessions in the cardiothoracic intensive care unit^20,35–37^. Populations in the cardiothoracic ICU who experience pre-surgical frailty or functional impairment may be at increased risk of complications following the initiation of well-known ICU medications impacting utilization of physical therapy interventions^38,39^. How these delays and complications impact patient treatment is difficult to determine but may relate to outcomes for distinct diagnoses. Identifying current delivery of physical therapy treatment in this population supports opportunities to redirect or shift staff responsibilities, improve clinical care and focus physical therapy treatment on those patients with greatest need^40,41^.

The purpose of this study is to determine existing mobilization practices as measured by timing and amount of physical therapy in patients with cardiac and respiratory problems requiring intervention in the cardiothoracic ICU (CT ICU). In addition, we were interested in differences in the time to first physical therapy evaluation and treatment for select patient surgical procedures and medical diagnoses, considering the level of patient complexity in this population, which has not previously been addressed. A comparison of hospital and CT ICU LOS between select patient subgroups is warranted to identify potential opportunities for physical therapists to promote more timely, efficient, and cost-effective physical therapy delivery for the distinct patient populations. This includes determining that associations exist between LOS in this patient population and timing and amount of physical therapy.

## Methods

### Design and Participants

We performed a retrospective, cross-sectional study of patients in the CT ICU at University of Kentucky (UK) HealthCare from March 2012 to May 2015 who received physical therapy evaluation and/or treatment as part of the standard of care. The UK Institutional Review Board approved this study. We accessed data via the UK Center for Clinical and Translational Science (CCTS) searching Soft Med and Avega programs for hospital data. Our population (n = 2,568) was based on individual patient encounters for all patients ≥18 years of age. We excluded pregnant women and prisoners from our population. Our patient population was divided into subgroups based on the highest volume procedures or diagnoses in the CT ICU during the study timeframe defined by the International Classification of Diseases, Ninth Edition (ICD-9)^42^. This includes patients post coronary artery bypass graft (CABG, n = 638), post valve replacement surgery (Valve, n = 339), post ventricular assist device implantation (VAD, n = 53), requiring extracorporeal membrane oxygenation (ECMO, n = 27), diagnosed with respiratory failure (mechanical ventilation >96 hours, n = 186), post heart transplantation (Heart transplant, n = 35), and post lung transplantation (Lung transplant, n = 32). All other patient procedures and diagnoses are included in the entire cardiothoracic patient population during the study time frame (n = 1258).

Patients received physical therapy evaluation and treatment based on consultation from the primary physician service line (e.g. Cardiology, Thoracic surgery, Cardiothoracic surgery, etc.) providing care for the patient during the hospitalization. At UK HealthCare, physical therapists have 24 hours after receiving notice of consultation to provide an initial evaluation or communicate to the medical team that physical therapy evaluation and treatment is deferred due to medical reasons. To identify patients who received physical therapy evaluation and treatment provided by either a physical therapist or physical therapist assistant at this facility, we used standard current procedural terminology (CPT) codes. The specific CPT codes used reflect physical therapy evaluation (97001), re-evaluation (97002), and other common CPT codes that describe rehabilitation treatment most frequently used by physical therapist and physical therapist assistant staff at this facility: therapeutic activities (97530), therapeutic exercise (97110), neuromuscular re-education (97112), and gait training (97116)^43^. This method allowed us to determine both mean days of physical therapy treatment and time to first physical therapy evaluation in the CT ICU or hospital facility. Time to first physical therapy evaluation in the CT ICU and hospital was determined using the date/time of admission in the CT ICU or hospital then identifying the date/time of the first physical therapy evaluation using the evaluation CPT code. Any date with a physical therapy CPT code entered for a patient was considered a day of physical therapy evaluation, re-evaluation or treatment. No patient encounters in this dataset were found to not receive some form of physical therapy evaluation and/or treatment during the hospitalization.

Using a retrospective review of the data limited our assessment of patient or disease severity in this population as we lacked access to data commonly collected such as APACHE II scores. However, we were able to determine a measure of patient complexity using Charlson Comorbidity Index (CCI) scores. Using CCI scores to express comorbidity has been a common method to control for confounding in epidemiologic studies and scores are used in predicting health outcomes, healthcare utilization, and healthcare expenditures^44^. Examining the CCI scores allows us to account for ICU treatment complexity required in the medical care of patients in the study population. The index lists 19 comorbid conditions, each assigned a weight from one to six. The CCI is the sum of the weights for all conditions for each patient^44^. We used the five digit enhanced ICD-9-CM (Clinical Modification) codes to determine CCI scores based on the algorithm developed by Quan H, *et al*.^45^.

The independent variables of interest for this analysis were mean days of physical therapy treatment during hospitalization (including in the CT ICU and after transfer or admission to the floor), means days requiring mechanical ventilation, time to first physical therapy evaluation (in the CT ICU or on other units within the hospital) in days, comorbidity score, and age during hospitalization. We distinguished between time to first physical therapy evaluation in the CT ICU as well as within the hospital facility, as patients may admit to other units before transfer to the CT ICU or may require physical therapy evaluation and treatment prior to surgery. The dependent variables of interest include CT ICU LOS and hospital LOS. Hospital LOS, measured in days, is used as an outcome measure of efficiency and resource utilization in hospital services. It is ultimately a measure of hospital quality, processes of care and access to healthcare^46^. Previous literature suggests that greater patient mobilization and physical therapy treatment provided during critical illness is associated with decreased hospital length of stay^9,47^.

All data were analyzed using JMP®, Version 12. SAS Institute Inc., Cary, NC, 2015. Initial descriptive statistics were calculated. The procedure and/or diagnoses were dummy coded for each subgroup in the patient population for comparison. One way analyses of variance (ANOVA) was performed where comorbidity score was not a significant factor between groups, and analyses of covariance (ANCOVA), where comorbidity was a significant factor between groups, to compare all variables except for age for the following populations: CABG, Valve, VAD, heart transplant or lung transplant, ECMO and respiratory failure. Pairwise multiple comparison procedures were performed using LSD method. Given our large sample size and our understanding of potential bias in our data, patients not included in any or included in more than one subgroup were excluded from the ANOVA and ANCOVA analysis. We had 1258 patients with greater than one or none of the diagnosis or surgical procedure during the study timeframe. Multivariate linear regression equations were estimated to examine the association between the independent variables and the dependent variables, using specification tests to rule out omitted variable bias. After performing a correlational matrix of the independent variables, we determined low correlation between variables with the exception of mean days of physical therapy and mean days of mechanical ventilation (0.49). Variance inflation factors suggested minimal risk of multicollinearity in the model. A logarithmic transformation of the dependent LOS variable (hospital and CT ICU LOS) was performed to create normal distribution and improve model fit. Multivariate semi-logarithmic regression models were estimated using the same independent variables in the linear regression models.

### Ethics Approval and Consent to participate

We received ethics approval from the University of Kentucky Institutional Review Board, Office of Research Integrity, IRB #15-0276-P1H. Informed consent was waived since the study was a retrospective review of data. All research procedures were carried out in accordance with relevant guidelines and regulations.

### Data availability

The datasets generated and analyzed during the current study are not publicly available due to restrictions from the healthcare system under study but are available from the corresponding author on reasonable request.

## Results

### Descriptive Statistics

Table 1 includes the demographic information and descriptive statistics of our patient population including comorbidity for each patient subgroup (Table 1). Racial and ethnic categories were not described since analysis of the data revealed the population to be predominately homogenous (Caucasian).

**Table 1:** Descriptive Statistics.

Procedure Group	Age	Gender, Male	Comorbidity Score
All of CT ICU	59.9 ± 14.6 (1310)	896 (1,310)	4.4 ± 2.9 (1230)
CABG	61.9 ± 10.3 (638)	490 (638)	4.0 ± 2.8 (592)
Valve	60.7 ± 19.5 (339)	190 (339)	3.9 ± 2.7 (314)
VAD	53.8 ± 10.6 (53)	43 (53)	5.2 ± 2.6 (53)
Heart Transplant	48.5 ± 14.9 (35)	22 (35)	5.0 ± 2.7 (35)
Lung Transplant	52.4 ± 14.7 (32)	23 (32)	5.7 ± 3.7 (32)
ECMO	53.3 ± 16.4 (27)	13 (27)	4.6 ± 2.9 (27)
Respiratory Failure	57.9 ± 15.1 (186)	115 (186)	5.9 ± 3.2 (177)

Values are means ± Standard deviation (n).

### Differences between Distinct Patient Subgroups

Analysis of variance or analysis of covariance if more appropriate indicates differences among certain patient subgroups for all variables other than age are greater than would be expected by random chance. See Tables 2–4 for all ANOVA results.

**Table 2:** ANOVA or ANCOVA Comparison of mean days of PT and days to first PT in the hospital and CT ICU between groups.

Procedure Groups	Mean days of PT (*)	Days to first PT in hospital	Days to first PT in the ICU
CABG	3.6 ± 2.6^3,4,5,6,7^ (638)	3.1 ± 2.6^2,3,4,6,7^ (638)	1.5 ± 1.1^4,6,7^ (638)
Valve	4.1 ± 3.2^3,4,5,6,7^ (339)	4.0 ± 6.0^1,3,4,7^ (339)	1.5 ± 1.0^4,6,7^ (339)
VAD	7.8 ± 6.9^1,2,7^ (53)	6.6 ± 4.9^1,2,4,5,7^ (53)	1.9 ± 1.2^4,6,7^ (53)
Heart transplant	7.7 ± 11.1^1,2,7^ (35)	10.3 ± 13.8^1,2,3,5,6^ (35)	2.8 ± 5.7^1,2,5,7^ (35)
Lung transplant	8.8 ± 6.1^1,2,7^ (32)	3.2 ± 2.2^3,4,5,7^ (32)	1.3 ± 1.0^4,6,7^ (32)
ECMO	8.1 ± 8.9^1,2,7^ (27)	5.8 ± 7.6^1,4,7^ (27)	3.0 ± 2.7^1,2,5,7^ (25)
Respiratory Failure	12.1 ± 16.4^1,2,3,4,5,6^ (186)	10.8 ± 7.8^1,2,3,5,6^ (186)	6.8 ± 6.0^1,2,3,4,5,6^ (183)
Not included in ANOVA			
All other CT ICU	6.1 ± 12.8 (1258)	4.6 ± 5.6 (1258)	2.8 ± 3.4 (1233)

1 = different from Coronary artery bypass graft, 2 = different from Valve replacement, 3 = different from Ventricular assist device, 4 = different from Heart transplant, 5 = different from Lung transplant, 6 = different from ECMO, 7 = different from Respiratory failure

Values are means ± (Standard deviation)

(*)ANCOVA was applied.

**Table 3:** ANOVA Comparison of CCI score and mean days on ventilation between groups.

Procedure Groups	CCI Score	Mean days of ventilation
CABG	4.0 ± 2.8^3,4,5,7^ (592)	1.0 ± 0.2^5,6,7^ (561)
Valve	3.9 ± 2.7^3,4,5,7^ (314)	1.0 ± 0.3^5,6,7^ (256)
VAD	5.2 ± 2.6^1,2^ (53)	1.1 ± 0.3^6,7^ (51)
Heart transplant	5.0 ± 2.7^1,2^ (35)	1.0 ± 0.4^5,6,7^ (28)
Lung transplant	5.7 ± 3.7^1,2^ (32)	1.3 ± 0.7^1,2,4^ (29)
ECMO	4.6 ± 2.9 ^7^ (27)	1.4 ± 0.7^1,2,3,4^ (16)
Respiratory Failure	5.9 ± 3.2^1,2,6^ (177)	1.6 ± 1.2^1,2,3,4^ (166)
Not included in ANOVA		
All other CT ICU	4.9 ± 3.3 (1197)	1.3 ± 0.8 (679)

1 = different from CABG, 2 = different from Valve, 3 = different from VAD, 4 = different from Heart transplant,

5 = different from Lung transplant, 6 = different from ECMO, 7 = different from Respiratory failure

Values are means ± Standard deviation (n).

**Table 4:** ANOVA Comparison of LOS in the hospital and CT ICU between groups.

Procedure Groups	Hospital LOS(*)	CT ICU LOS(*)
CABG	10.4 ± 6.9^2,3,4,5,6,7^ (638)	4.0 ± 2.6^3,4,5,6,7^ (638)
Valve	17.2 ± 16.9^1,3,4,7^ (339)	4.1 ± 2.9^3,4,5,6,7^ (339)
VAD	23.6 ± 16.5^1,2,7^ (53)	9.9 ± 12.4^1,2,7^ (53)
Heart transplant	25.6 ± 21.9^1,2,7^ (35)	11.3 ± 12.3^1,2,7^ (35)
Lung transplant	22.7 ± 15.5^1,7^ (32)	7.8 ± 5.9^1,2,7^ (32)
ECMO	22.8 ± 19.2^1,7^ (27)	12.1 ± 11.4^1,2,7^ (27)
Respiratory Failure	44.9 ± 43.9^1,2,3,4,5,6^ (186)	17.6 ± 22.9^1,2,3,4,5,6^ (186)
Not included in ANOVA		
All other CT ICU	19.6 ± 29.2 (1258)	8.6 ± 19.1 (1258)

1 = different from Coronary artery bypass graft, 2 = different from Valve replacement, 3 = different from Ventricular assist device, 4 = different from Heart transplant, 5 = different from Lung transplant, 6 = different from ECMO, 7 = different from Respiratory failure

Values are means ± Standard deviation (n) (*) ANCOVA was applied.

Differences in mean days of physical therapy and time to first physical therapy evaluation were observed for the subgroups (Table 2). Patients post CABG and Valve surgery had fewer mean days of physical therapy than all other subgroups. Patients post heart and lung transplant both had an average of 8 days of physical therapy and this was greater than patients post CABG and Valve surgery and fewer than patients diagnosed with respiratory failure. Days to first PT evaluation show patient procedure or diagnosis influenced initiation of PT in the hospital and CT ICU (Table 2). Patients with respiratory failure had on average more days until the first PT session in the ICU than all the other subgroups. Patients requiring more invasive cardiac surgery or support (patients post heart transplant and who required ECMO) had greater delay in days to first PT in the CT ICU compared to patients requiring less invasive cardiac surgery (post CABG, Valve, and VAD) (Table 2). Patients with heart transplant and respiratory failure had the greatest mean days to PT in the hospital. Data for patients not included in the subgroups were added for comparison, but were not part of the ANOVA analysis.

Comorbidity score was found to be significantly different between select patient subgroups (Table 3). Patients post CABG and Valve surgery showed the lowest CCI scores in the population and were different from all subgroups other than patients requiring ECMO (Table 3). Patients with respiratory failure had a much larger CCI score compared to patients requiring ECMO (Table 3). Mean days of mechanical ventilation were also found to be different between select subgroups. However, no distinguishable difference was found between patients post lung transplant, requiring ECMO and with respiratory failure (Table 3). Data for patients not included in the subgroups were added for comparison, but were not part of the ANOVA analysis.

Table 4 shows differences in hospital and CT ICU LOS between the distinct patient subgroups. There is no difference between the hospital or CT ICU LOS for patients post VAD, heart transplant, lung transplant, and requiring ECMO (Table 4). Interestingly, patients post CABG or post Valve surgery have shorter CT ICU and hospital LOS overall, yet statistically significant differences exist between these two patient subgroups for hospital LOS (Table 4). Patients with respiratory failure are significantly different than all other subgroups, showing significant differences in hospital and CT ICU LOS compared to all other subgroups (Table 4). Data for patients not included in the subgroups were added for comparison, but were not part of the ANOVA analysis.

### Regression Results

We ran a semi logarithmic regression model to determine if timing and amount of physical therapy is associated with hospital and CT ICU LOS and how strongly the variables in the model explain hospital and CT ICU LOS for the patient subgroups (Tables 5 and 6). Using the variables time to first PT in the ICU, CCI score, mean days of PT, and mean days of mechanical ventilation showed strong and large variances in hospital (Table 5) and CT ICU LOS (Table 6). When using this semi logarithmic regression model for hospital LOS in each sub population of CT ICU patients, all patient subgroups showed adjusted R^2^ greater than 0.49 (49%) (Table 5). Nearly 50% of the variability of hospital LOS in these patient subgroups can be explained by the independent variables. For example, in patients post lung transplant, this model explained 74% of the variability in hospital LOS.

**Table 5:** Regression Results for Hospital Length of Stay (LOS).

Hospital LOS	All other in CT ICU Population		CABG		Valve		VAD		ECMO		Respiratory Failure		Heart Transplant		Lung Transplant	
	Beta	SE	Beta	SE	Beta	SE	Beta	SE	Beta	SE	Beta	SE	Beta	SE	Beta	SE
Days to PT	0.060^^^	0.004	0.049	0.007	0.067^^^	0.017	0.015	0.023	0.017	0.014	0.007	0.006	0.054^^^	0.022	0.072^^^	0.034
CCI	0.050^^^	0.005	0.036^^^	0.005	0.058^^^	0.014	0.023	0.021	0.056^^^	0.026	0.033^^^	0.010	0.009	0.045	0.008	0.022
Mean Days of PT	0.045^^^	0.020	0.106^^^	0.004	065^^^	0.005	0.038^^^	0.005	0.026^^^	0.003	0.026^^^	0.002	0.033^^^	0.007	0.265^^^	0.003
Mean Days of Vent	0.202^^^	0.025	0.100^^^	0.036	0.199^^^	0.071	0.245^^^	0.094	0.014	0.074	0.102^^^	0.029	0.301	0.187	0.191	0.105
Age	−0.008^^^	0.001	0.000	0.001	−0.145^^^	0.002	−0.003	0.005	−0.006	0.005	−0.006^^^	0.002	−0.005	0.008	−0.003	0.006
constant	2.16^^^	0.068	1.44	0.091	2.57^^^	0.146	2.32	0.318	3.05	0.275	3.15	0.138	2.47	0.432	2.53	0.317
Adjusted R^2^	0.564		0.673		0.520		0.701		0.492		0.540		0.670		0.740	
Root MSE	0.576		0.340		0.616		0.436		0.618		0.517		0.602		0.455	

^^^p < 0.05.

**Table 6:** Regression Results for Cardiothoracic Intensive Care Unit Length of Stay (CT ICU LOS).

CT ICU LOS	All other in CT ICU Population		CABG		Valve		VAD		ECMO		Respiratory Failure		Heart Transplant		Lung Transplant	
	Beta	SE	Beta	SE	Beta	SE	Beta	SE	Beta	SE	Beta	SE	Beta	SE	Beta	SE
Days to 1^st^ PT	0.046^^^	0.005	0.093^^^	0.011	0.088^^^	0.016	0.090^^^	0.022	−0.003	0.014	−0.030^^^	0.009	0.072^^^	0.020	0.130^^^	0.364
CCI	0.028^^^	0.006	0.017^^^	0.007	0.030^^^	0.014	0.007	0.020	0.020	0.025	0.005	0.016	−0.014	0.041	0.021	0.024
Mean Days of PT	0.045^^^	0.002	0.073^^^	0.006	0.052^^^	0.005	0.052^^^	0.005	0.026^^^	0.003	0.026^^^	0.003	0.033^^^	0.007	0.032^^^	0.004
Mean Days of Vent	0.283^^^	0.028	0.398^^^	0.051	0.434^^^	0.069	0.319^^^	0.092	0.082	0.071	0.086	0.046	0.519^^^	0.169	0.368^^^	0.114
Age	0.00	0.001	0.005^^^	0.002	0.005^^^	0.002	0.000	0.005	−0.002	0.005	0.000	0.003	0.002	0.007	−0.015^^^	0.007
constant	0.717	0.076	0.055	0.128	0.168	0.142	1.10	0.311	2.47	0.266	2.29	0.217	1.06	0.392	1.72	0.343
Adjusted R^2^	0.483		0.484		0.483		0.820		0.550		0.361		0.780		0.810	
Root MSE	0.651		0.477		0.600		0.426		0.599		0.812		0.545		0.494	

^^^p < 0.05.

The variables most frequently determined to be statistically significant for each subgroup model were time to first PT in the CT ICU, mean days of PT, and mean days of mechanical ventilation (Tables 5 and 6). Using this model to explain CT ICU LOS in each patient subgroup showed smaller variance for certain subgroups (CABG, Valve and respiratory failure) and larger variance in others (VAD, heart transplant and lung transplant) (Table 6). The same variables for timing and amount of physical therapy demonstrated strong associations with CT ICU LOS (Table 6).

## Discussion

We have successfully investigated variables related to timing and amount of physical therapy for select patient subgroups in a CT ICU. Our results indicate that timing and amount of physical therapy is more strongly correlated to procedure or medical intervention during hospitalization than to patient comorbidity. In addition, time to first PT session, mean days of PT, CCI, mean days of ventilation, and age explained large variability of hospital LOS for patients in the subgroups examined and of these, mean days of mechanical ventilation show the highest correlation.

Patients in the CT ICU experience shorter time to first PT evaluation and treatment compared to the time to first PT evaluation and treatment while on a hospital floor unit. This may reflect a practice culture signifying a strong commitment to mobilization of appropriate patients in this hospital’s CT ICU. At this facility, there has been a gradual increase in physical therapist or physical therapist assistant presence in the CT ICU and cardiothoracic floor units over the study timeframe. We hypothesize that barriers to physical therapy at hospital admission or in the Emergency Department exist that prevent CT ICU patients from receiving earlier PT evaluation and treatment before they are admitted to the CT ICU. Patients experiencing a delay in transfer to the CT ICU where PT evaluation and treatment appears to begin sooner may be at higher risk for immobility and development of weakness prior to surgical intervention. Immobility and functional decline during hospitalization has negative consequences, which can include prolonged LOS^11,31^. Further study is required to determine whether patients admitted directly to the CT ICU or more quickly receiving PT evaluation and treatment on other units could improve patient outcomes after CT surgery or mechanical ventilation through pre-habilitation.

Patients post lung transplantation experienced the shortest time to PT evaluation in the CT ICU, yet it was not significantly different from patients post VAD, CABG and Valve surgery. These patients have the second highest CCI score in the data, which was found to be different from those patients post CABG and Valve surgery. Interestingly, patients post lung transplant had mechanical ventilation needs that differed from patients post CABG, Valve or heart transplantation. Clearly, additional factors other than CCI and mechanical ventilation were influencing timing and amount of physical therapy treatment in these patients. Overall, studies have indicated that survival post lung transplantation is suboptimal with patients at high risk for adverse outcomes such as renal failure, malignancy, infection and poor quality of life^48,49^. The Center for Medicare and Medicaid Services closely monitors all transplant program outcomes, specifically for observed and expected mortality^50^. Programs are required to self-report their quality data to the Organ Procurement and Transplantation Network. For this reason, lung transplant program outcomes are scrutinized and program sustainability is an ongoing high priority for hospitals^51^. Our findings indicate that high risk post lung transplant patients receive physical therapy during acute critical illness and physical therapy ICU staff may have prioritized patients with lung transplantation. This contrasts starkly to the delay patients requiring more invasive cardiac intervention (VAD and heart transplant) experience in time to first PT evaluation in the hospital and in the CT ICU (heart transplant patients only). A reason for delay in time to first PT evaluation in patients post heart transplant or VAD implantation is beyond the scope of this manuscript, but we hypothesize it was related to delayed chest closure and initial cardiac hemodynamic instability after surgery. The reasons for open chest management are varied^52^, and have been associated with an increase in LOS^53^ and mechanical ventilation^52^. Despite the shorter time to first PT evaluation and treatment in patients post lung transplant, there was no statistically significant difference in hospital or CT ICU LOS between patients post lung transplant, post heart transplant, or post VAD. Similarly, mean days of PT during hospitalization were not significantly different between those three groups. Additional institutional factors that may have confounded the LOS data are individual physician practices in discharge from the ICU to the hospital floor and limitations in bed availability on hospital floor units. Future physical therapy research in the CT ICU should address the most effective and efficacious physical therapy interventions, including intensity, frequency and dosage for these subgroups to determine impact on LOS.

CT ICU LOS was not statistically significantly different for patients post CABG or Valve replacement surgery which may be explained by the similarities in average CCI score and mechanical ventilation requirements. However, overall hospital LOS difference suggests that once these patients leave the CT ICU, LOS trajectory is altered. One explanation may be related to mobility after patients are transferred to the floor. Critical care patients experience low mobility once transferred to floor units including delays in mobility or physical therapy treatment after transfer and in some cases regression in mobility level^54–56^. There may be differences in mobility level of patients post CABG and Valve surgery after transfer off the CT ICU, or differences in complication rates unable to be determined from this study.

Regression results showed that variables related to timing and amount of physical therapy (time to first PT, mean days of PT and mean days of mechanical ventilation) were highly correlated with hospital, and especially CT ICU LOS for patients post VAD, heart transplant and lung transplant. Additionally, the variables mean days of mechanical ventilation and mean days of PT were statistically significant in the three subgroup models with the largest Beta values. Mean days of mechanical ventilation explain a greater percentage of variability in hospital and CT ICU LOS. Enhanced management of mechanical ventilation and education about Acute Respiratory Distress Syndrome (ARDS) may be indicated. In a recent study of 50 ICUs nationally, clinician recognition of ARDS was poor as was the implementation of evidence-based therapy^57^. Frequently cited barriers to mobilization in the general ICU population include sedation and endotracheal intubation^17,19^. Examples of processes that streamline physical therapy consultation and mobility in the ICU for patients requiring mechanical ventilation include timing and coordination of spontaneous breathing trials with physical therapy assessments and changing sedation practices^19^ or sedation holidays in patients requiring prolonged mechanical ventilation. This is important knowledge for clinicians in the ICU because it indicates that clinical practice can be changed to influence LOS in these populations.

There are several limitations with this study. Using retrospective, administrative data limits the causality of our conclusions. The validity and reliability of ICD-9 CM diagnosis coding in administrative studies has been questioned, as the ICD-9 CM system was not designed for research purposes^58^. There are many factors related to the accuracy of coding including physician documentation, coder training and experience and ICD-9 code ambiguity^42,58^. When measuring physical therapy, we considered one day of PT as any day where patients’ had a physical therapy CPT code documented yet we are unable to determine the quantity of the CPT code (minutes) or which code was documented. Thus, we were unable to identify the amount of therapy or level of mobility achieved for patients each day. Without more description of the physical therapy activities or the intensity of physical therapy patients received, we were limited in determining factors associated with physical therapy practice. However, strong correlations were found in the variables chosen for this study.

This study represents a comprehensive analysis of variables related to physical therapy evaluation and treatment during CT ICU hospitalization. We found that timing and amount of physical therapy in patients with cardiac and respiratory illness differs more based on procedure required during hospitalization than on patient comorbidity. Timing and amount of physical therapy is associated with hospital and CT ICU LOS in this patient population. The data provided here indicate that variables such as time to first physical therapy evaluation in the ICU and the hospital, and mean days of physical therapy need to be considered in future prospective studies.
